# Supplementary material for: Nutrient Availability and Biofilm Polysaccharide Shape the Bacillaene-Dependent Antagonism of Bacillus subtilis against Salmonella Typhimurium
Source: Microbiol Spectr. 2022 Nov 7;10(6):e01836-22. doi: 10.1128/spectrum.01836-22 (PMC9769773; doi:10.1128/spectrum.01836-22)
Supplement: Supplemental file 1 — Supplemental material. Download spectrum.01836-22-s0001.pdf, PDF file, 1.5 MB [file spectrum.01836-22-s0001.pdf]

Supplemental material

Nutrient availability and biofilm polysaccharide shape the bacillaene - dependent antagonism of *Bacillus subtilis* against *Salmonella* Typhimurium

Eli Podnar[1], Andi Erega[1], Tjaša Danevčič[1], Eva Kovačec[1], Bram Lories[2], Hans Steenackers[2], Ines Mandic-Mulec[1,3*]

[1]Department of Microbiology, Biotechnical University, University of Ljubljana, Ljubljana, Slovenia

[2]Department of Microbial and Molecular Systems, Centre of Microbial and Plant Genetics (CMPG), KU Leuven, Leuven, Belgium

[3]Chair of Microprocess Engineering and Technology - COMPETE, University of Ljubljana, Ljubljana, Slovenia

Running title: Nutrient dependent *Bacillus* effects on *Salmonella*

*Corresponding author: Ines Mandic Mulec

ines.mandicmulec@bf.uni-lj.si

This PDF file includes:

Figures S1 to S4

Tables S1 and S2

Supplemental Figures

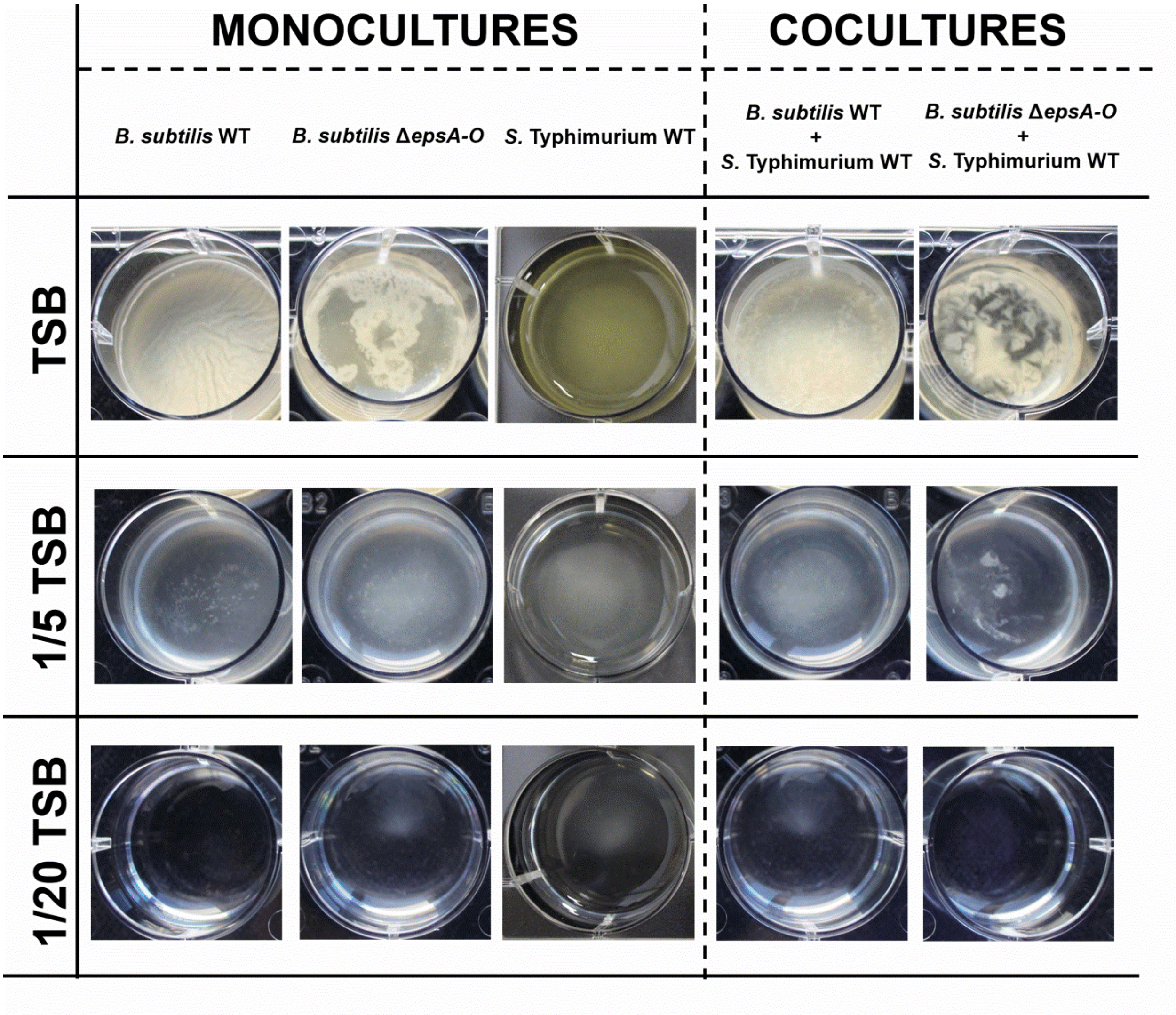

Fig. S1. Biofilms of *B. subtilis* PS-216 WT (BM1097) strain, *B. subtilis* PS-216 Δ*epsA-O* mutant (BM1310) and *S.* Typhimurium SL1344 strains grown statically in TSB, 1/5 TSB and 1/20 TSB medium at 37 °C for 24 hours.

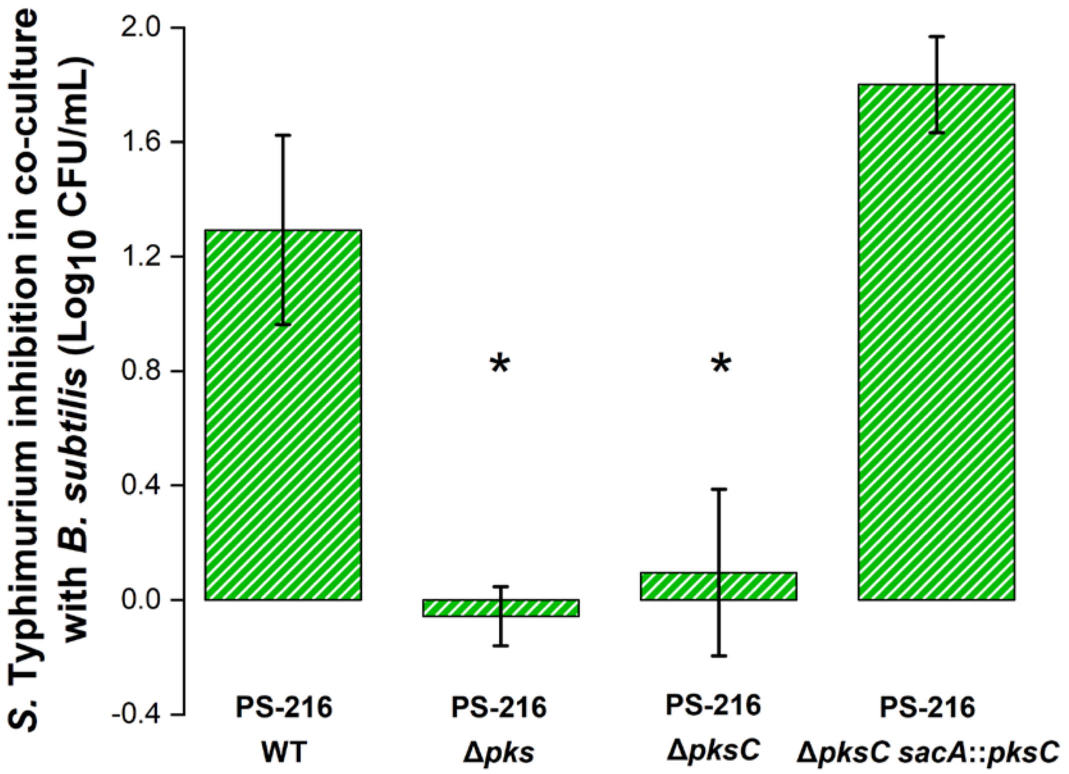

Fig. S2. Growth inhibition of *S.* Typhimurium SL1344 GFP in coculture with the *B. subtilis* PS-216 WT strain (BM1097), *B. subtilis* PS-216 Δ*pks* (BM1876), *B. subtilis* PS-216 Δ*pksC* (BM1957) and *B. subtilis* PS-216 Δ*pksC sacA::pksC* (BM1959), which also carried the *pksC* gene integrated into the *sacA* locus. Measurements of CFU/mL were performed after 24 h of coincubation in static conditions in TSB medium at 37 ^{o}C. Data sets represent mean values with the standard deviation of three biological replicates. A Student's t-test was used to determine statistically significant differences ($p < 0.05$) to WT strain, which are marked with *.

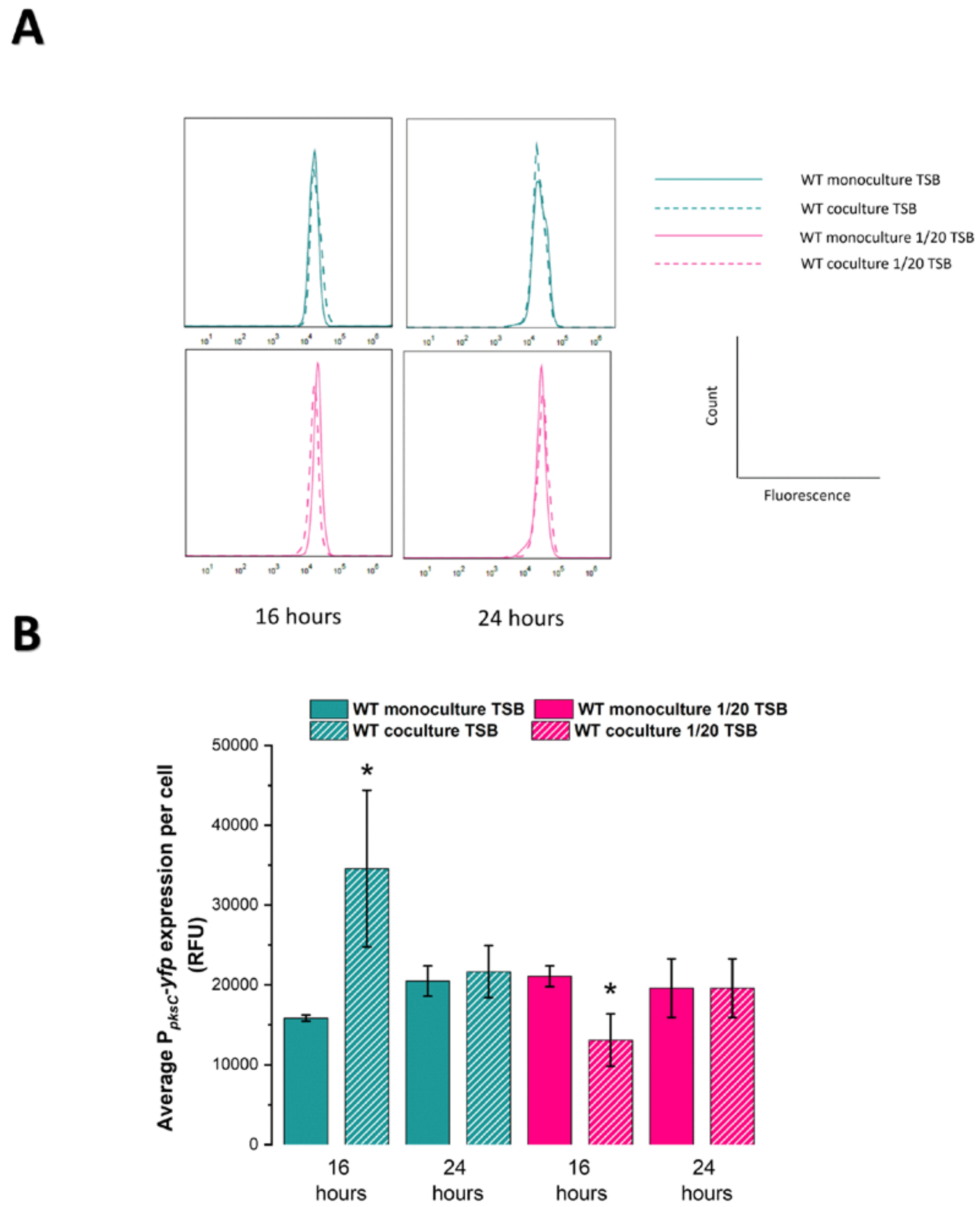

Fig. S3. Single cell expression analysis of P_{pksC}-*yfp* promoter activity in *B. subtilis* BM1884 strain during static growth in TSB and 1/20 TSB medium in monoculture and coculture after 16 hours and 24 hours of incubation at 37 °C. A) FACS profiles of *B. subtilis* P_{pksC}-*yfp* expressing cells. B) Average fluorescence intensity of P_{pksC}-*yfp* expressing cells. A Student's t-test was used to determine statistically significant differences ($p < 0.05$) to monoculture strain, which are marked with *.

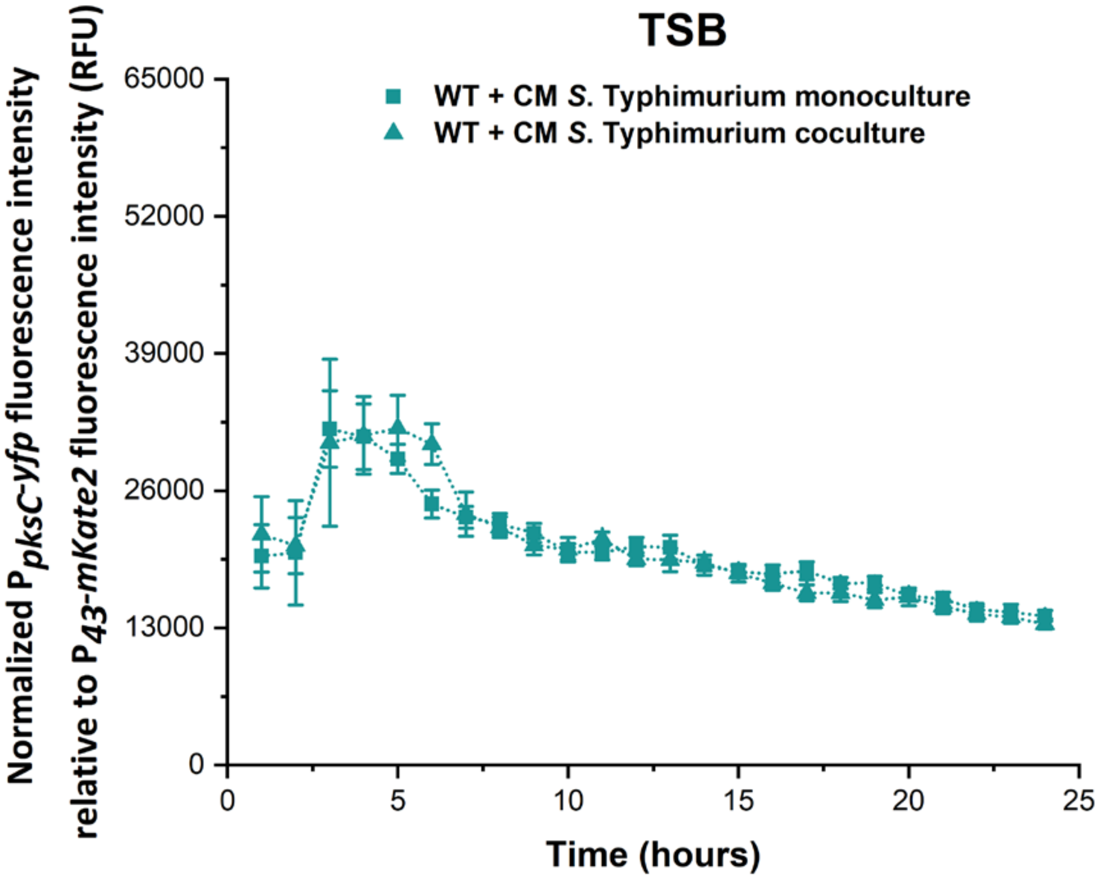

Fig. S4. Transcriptional activity of P_{pksC}-*yfp* promoter of the *B. subtilis* BM1884 strain supplemented with conditioned medium (CM) of *S*. Typhimurium SL1344 monoculture and coculture. Measurements of P_{pksC}-*yfp* promoter activities were performed in TSB medium every half an hour (only data points measured every hour are shown for clarity). Results are presented as relative units using a constitutively expressed P_{43}-*mKate2* promoter as a proxy for *B. subtilis* biomass.

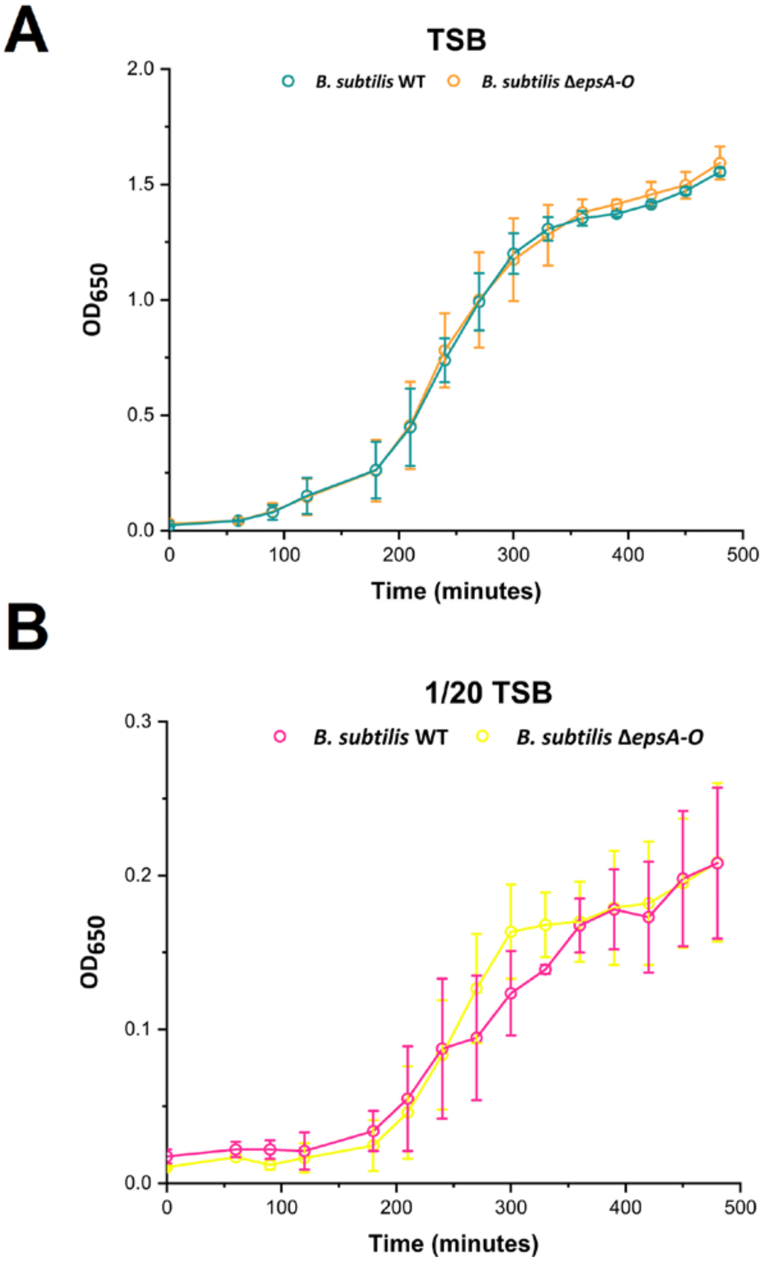

Fig. S5. Growth curves of *B. subtilis* PS-216 WT strain (BM1097) and *B. subtilis* PS-216 Δ*epsA-O* mutant (BM1310) in TSB medium (A) and 1/20 TSB medium (B). Bacterial strains were grown in monoculture in TSB and 1/20 TSB media for 8 hours in shaking conditions at 37 °C. Measurements were performed every half an hour and means with standard deviations of three biological replicates are shown.

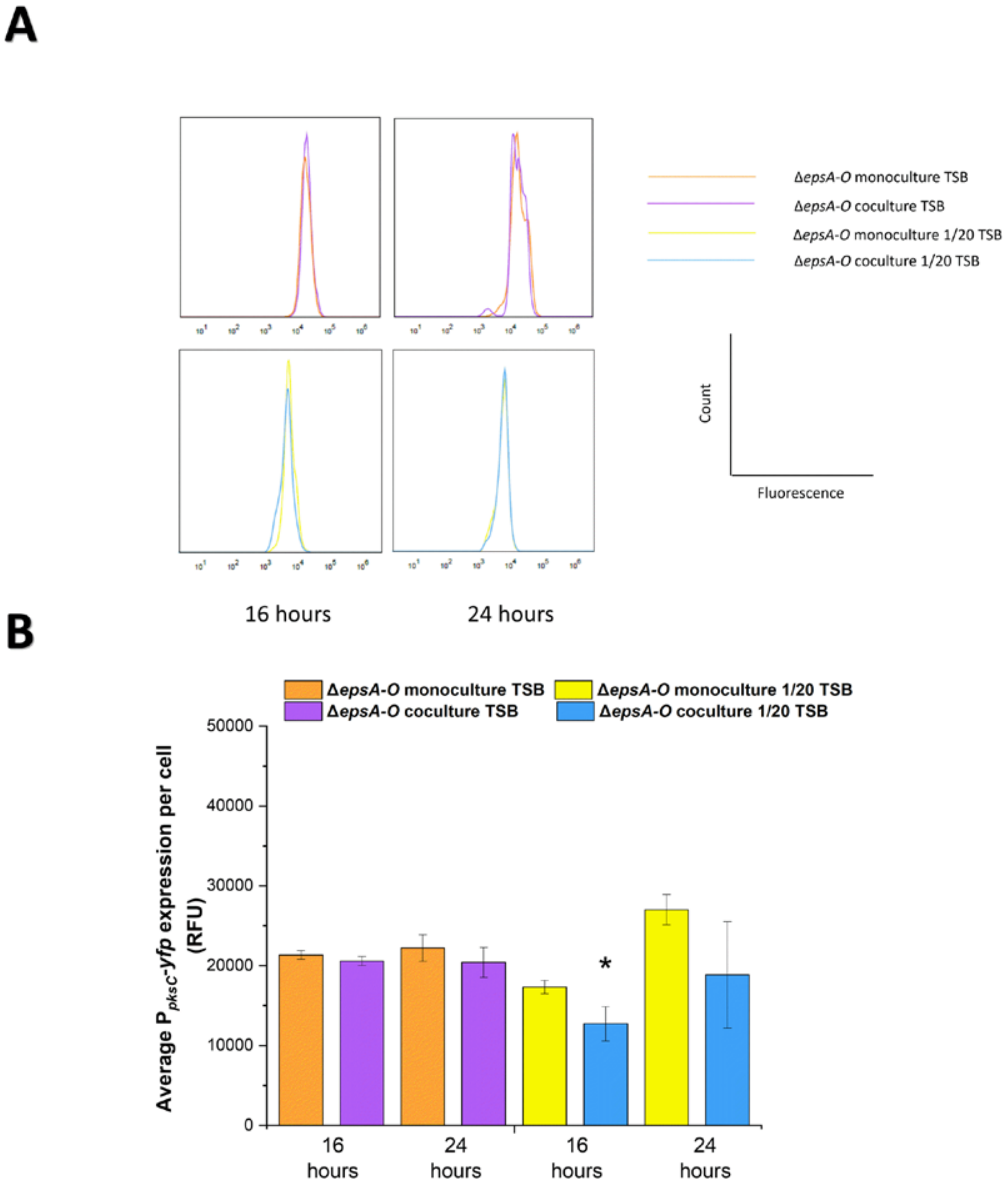

Fig. S6. Single cell expression analysis of P_{pksC}-*yfp* promoter activity in *B. subtilis* BM1901 strain during static growth in TSB and 1/20 TSB medium in monoculture and coculture after 16 hours and 24 hours of incubation at 37 °C. A) FACS profiles of *B. subtilis* P_{pksC}-*yfp* expressing cells. B) Average fluorescence intensity of P_{pksC}-*yfp* expressing cells. A Student's t-test was used to determine statistically significant differences ($p < 0.05$) to monoculture strain, which are marked with *.

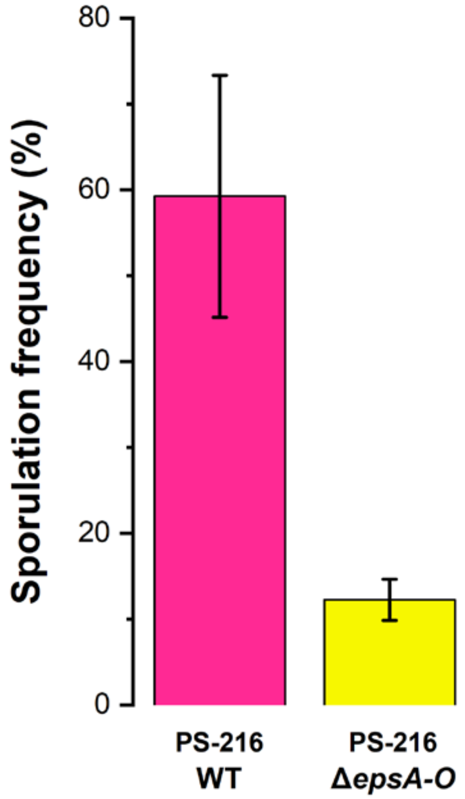

Fig. S7. The sporulation frequency of *B. subtilis* PS-216 WT strain (BM1097) and PS-216 Δ*epsA-O* mutant (BM1310) after 24 hours of incubation in 1/20 TSB medium.

Supplemental Tables

Table S1. Oligonucleotides used in this study

Oligonucleotides name	Sequence 5' → 3'	Reference
pksX1	GAATACGTAGCGTACAGCAAGCC	(1)
pksX4	AAACGGTTCGGAGCCACATATCC	(1)
5pL-pksC	CTGGTCCATCGTTTAAGAACTTTAC	(2)
5pR-pksC	GATCTTCTTCACCAGAAATGACG	(2)
pksC compl-F (HindIII)	CCCAAGCTTCCATTCGATAAAGGAT	This work
pksC compl-R (BamHI)	CGCGGATCCTCTGTGTTTAGACCTGATTG	This work
pC-F(EcoRI)	TTAGAATTCCCATTCGATAAAGGAT	(3)
pC-R(HindIII)	TATGAAGCTTGATTAGTAGATGTGTTTCAC	(3)

Table S2. Plasmids used in this study

Plasmid name	Background	Genotype	Reference
pSac-Cm	*E. coli* DH5α	*sacA*::*cat* (Cm, Amp)	(4)
pEM1112	*E. coli* DH5α	*sacA*::*pksC* (Cm, Amp)	This work
pKM3	*E. coli* DH5α	*amyE*::P_{spoIIQ}-*yfp* (Sp, Amp)	(5)
pEM1108	*E. coli* DH5α	*amyE*::P_{pksC}-*yfp* (Sp, Amp)	This work
pMS17	*E. coli* DH5α	*sacA*::P_{43}-*mKate2* (Kn, Amp)	(6)

References

1. Straight PD, Fischbach MA, Walsh CT, Rudner DZ, Kolter R. 2007. A singular enzymatic megacomplex from *Bacillus subtilis*. Proc Natl Acad Sci U S A 104:305–310.

2. Koo BM, Kritikos G, Farelli JD, Todor H, Tong K, Kimsey H, Wapinski I, Galardini M, Cabal A, Peters JM, Hachmann AB, Rudner DZ, Allen KN, Typas A, Gross CA. 2017. Construction and analysis of two genome-scale deletion libraries for *Bacillus subtilis*. Cell Syst 4:291-305.e7.

3. Vargas-Bautista C, Rahlwes K, Straight P. 2014. Bacterial competition reveals differential regulation of the *pks* genes by *Bacillus subtilis*. J Bacteriol 196:717–728.

4. Middleton R, Hofmeister A. 2004. New shuttle vectors for ectopic insertion of genes into *Bacillus subtilis*. Plasmid 51:238–245.

5. Burton BM, Marquis KA, Sullivan NL, Rapoport TA, Rudner DZ. 2007. The ATPase SpoIIIE transports DNA across fused septal membranes during sporulation in *Bacillus subtilis*. Cell 131:1301–1312.

6. Spacapan M, Danevčič T, Stefanic P, Porter M, Stanley-Wall NR, Mandic-Mulec I. 2020. The ComX quorum sensing peptide of *Bacillus subtilis* affects biofilm formation negatively and sporulation positively. Microorganisms 8:1131.
